# Interventions to promote home radon testing: A randomized clinical trial of a smartphone app vs. printed brochures

**DOI:** 10.1002/cam4.4988

**Published:** 2022-06-28

**Authors:** Soojung Kim, Tiffany Chiu, Marilyn G. Klug, David Schmitz, Gary G. Schwartz

**Affiliations:** ^1^ Department of Communication College of Arts and Sciences, University of North Dakota Grand Forks North Dakota USA; ^2^ Department of Education, Health & Behavior Studies College of Education & Human Development, University of North Dakota Grand Forks North Dakota USA; ^3^ Department of Population Health School of Medicine and Health Sciences, University of North Dakota Grand Forks North Dakota USA; ^4^ Department of Family and Community Medicine School of Medicine and Health Sciences, University of North Dakota Grand Forks North Dakota USA

**Keywords:** lung cancer, prevention, radon, risk communication, smartphone

## Abstract

Radon is a preventable cause of lung cancer, but the percentage of homes tested for radon is low. We previously developed a smartphone app that informs users about radon and allows them to request a free radon test. Here we conducted a randomized, controlled trial comparing the radon app versus printed brochures on radon knowledge, attitudes, and behaviors, including the proportion of participants requesting radon tests. Participants (N = 138) were undergraduates at a midwestern university. Data were analyzed by *t*‐tests, general linear models, and logistic regression. App users showed significantly greater increases in radon knowledge (*p* = 0.010) and self‐efficacy (*p* < 0.001) and requested tests three times more often than brochure recipients (41.4% vs. 13.2%, *p* < 0.001). However, the rate of test usage in each condition was low, ~3%. In conclusion, the radon app markedly outperformed brochures in increasing knowledge and requests for radon tests. Future work should focus on methods to increase test usage.

## Graphical abstract

We developed an app for smartphones to encourage radon testing of homes and compared it to print brochures in its ability to stimulate radon test requests.The radon app was markedly superior to brochures and increased test requests 3‐fold.

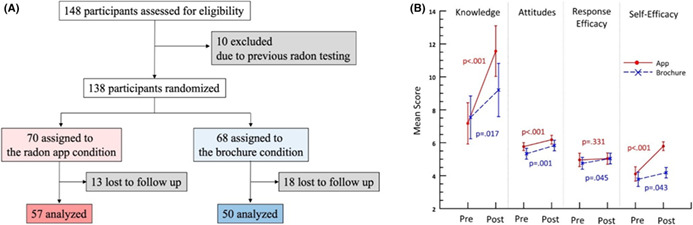

## 1 INTRODUCTION

Radon, an invisible gas produced from the natural decay of radioactive elements in rocks and soils, causes more than 21,000 lung cancer deaths annually in the U.S.^1^ Radon enters homes via cracks in the foundation and can accumulate indoors. Although 1 in 15 homes has radon levels that meet or exceed the threshold for remediation recommended by the EPA (4 pCi/L), the percentage of Americans who report testing their homes is low, ranging from 3% to 30%.^2^

Low radon testing rates might be due to the failure of standard communication methods, for example, printed brochures, to motivate individuals to obtain radon tests. For example, in a trial in Minnesota in which 250 participants received a brochure from their primary care provider and a coupon for a reduced price radon test, only 14% tested their homes after 1 year of follow‐up.^3^ A large Canadian study reported similar findings.^4^ Thus, alternate methods to promote radon testing are urgently needed.

Compared to information in printed formats, information in interactive formats, for example, smartphone applications (apps), often is more effective in increasing knowledge and promoting behavior change.^5^, ^6^, ^7^ We therefore developed a radon‐education app for smartphones.^8^ Here, we compared the performance of the radon app on individuals' radon knowledge, attitudes, and testing behaviors in a randomized controlled trial vs. the same information delivered via print brochures.

## 2 MATERIAL AND METHODS

### 2.1 Study design and sample

We used a 2‐arm, pretest‐posttest randomized controlled design. Participants were 148 undergraduates from the University of North Dakota in Grand Forks. Radon levels in Grand Forks are among the highest in the U.S.^9^, ^10^ The principal eligibility criteria were age of at least 18 years and smartphone ownership. Potential participants were excluded if they had tested for radon within the past 2 years.

Power calculations (Power Analysis and Sample Size Software 2020) indicated that a sample of 130 cases and controls could detect differences in proportions of 10% or greater and risk ratios of 1.2 or higher with a minimum power of 80% and α of 0.05.^11^

### 2.2 Procedure

Participants were students in an introductory communication class for which research participation (in one of several studies) was a requirement. Participants who chose to participate in this study completed pre‐ and post‐exposure surveys measuring radon knowledge, testing attitudes, and behavior. They were assigned either to the radon app (the experimental group) or brochure condition (usual care) via the randomization function in Qualtrics XM. The radon content in the app and brochure conditions was identical and differed only in the method of delivery.

Brochure participants received three EPA brochures over a three‐month period via U.S. mail: Basic Radon Facts (https://bit.ly/2S6ryFw), A Citizen's Guide to Radon (https://bit.ly/3l3FhcJ), and Consumer's Guide to Radon Reduction (https://bit.ly/3ibt83n). Each mailing included a pre‐paid postcard that could request a free radon test. Alternately, app participants installed the app on their smartphone and were asked to use it “occasionally” for 3 months. They could request a radon test at any time through the app. Bar codes on the radon tests enabled us to count the tests ordered and determine whether they had been returned to the laboratory.

Participants received extra course credit and a gift card in exchange for participation in the pre‐exposure (T1) and post‐exposure (T2) surveys. Informed consent was obtained via computer. IRB approval was obtained from the University of North Dakota (IRB # IRB0003245), and the trial was registered in ClinicalTrials.gov (ID: NCT04980521).

### 2.3 Measurement

*Radon knowledge* was measured by the number of accurate responses to 20 True‐False statements.^8^ *Attitudes about radon testing* were assessed by five, 7‐point semantic differential scales (e.g., “bad – good”).^12^ *Response efficacy*, that is, individuals' evaluation of the effectiveness of recommended behavioral advice, and *self‐efficacy*, individual's ability to follow through recommended behaviors, were each measured by two, 7‐point Likert scales.^8^, ^13^, ^14^ *Behavioral outcomes* were the percentage of participants who ordered free radon tests and the percentage that returned them to the laboratory.

### 2.4 Statistical analysis

Changes in knowledge, attitudes, response efficacy, and self‐efficacy were tested by paired, one‐tailed *t*‐tests. Differences in these changes between participants in the two conditions were tested with general linear models. Differences in the percent of participants who ordered a free radon test were tested by *Z* tests. Interactions between participants' attributes and the frequency of test requests were tested by logistic regression. We estimated relative risks from two‐by‐two tables stratified by participants' attributes.

## 3 RESULTS

**FIGURE 1:**
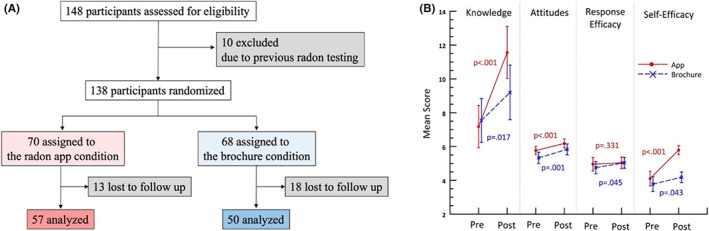
(A) Consort diagram showing randomization, exclusions, and losses to follow up in the radon app and brochure conditions. (B) Change in average respondent scores for radon app and brochure participants.

The Consort diagram is shown in Figure 1A. Of the 148 potential participants, 10 were ineligible due to previous radon testing. The remaining 138 participants were randomized to the two conditions. Thirty‐one (31) (31/148, 22.5%) were lost to follow‐up, 13/70 in the app and 18/68 in the brochure conditions (*p* = 0.13, Z test), leaving 57 participants in the app and 50 in the brochure condition. There were no significant differences in the groups post‐randomization with respect to sex (*p* = 0.960), age (*p* = 0.432), race/ethnicity (*p* = 0.879), income (*p* = 0.989), off‐campus versus on‐campus residence (*p* = 0.506), tobacco (*p* = 0.128), and/or e‐cigarette use (*p* = 0.236).

### 3.1 Participants' characteristics

The average age of participants was 20.4 (SD = 2.8). Eighty‐seven (63%) were female. Most self‐identified as White (*n* = 115, 83.3%). The majority (*n* = 77, 55.8%) lived on campus. Tobacco use was reported by 11 (8%) and e‐cigarettes by 28 (20.3%).

### 3.2 Scale construction and reliability statistics

To create *radon knowledge* index variables, correct responses to each statement were coded as 1 and incorrect responses or “Do not Know” as 0. Variables representing *radon testing attitudes, response efficacy*, and *self‐efficacy* were created by averaging scales for each variable. All reliability statistics (Cronbach's α) among measurement scales were higher than 0.70.

### 3.3 Comparing the app with brochures on knowledge, testing attitudes, and efficacy

Figure 1B shows the average scores for pre‐ and post‐exposure surveys by condition. Among radon app participants, radon knowledge (*p* < 0.001), radon testing attitudes (*p* < 0.001), and self‐efficacy (*p* < 0.001) increased significantly between T1 and T2. Among brochure participants, radon knowledge (*p* = 0.017), radon testing attitudes (*p* = 0.001), response efficacy (*p* = 0.045), and self‐efficacy (*p* = 0.043) increased between T1 and T2. There were significant interactions with the experimental condition and radon knowledge (*p* = 0.010) and self‐efficacy (*p* < 0.001).

### 3.4 Comparing the app with brochures on ordering and utilizing free radon tests

Among radon app participants, 29/70 (41.4%) ordered a test, versus 9/68 (13.2%) in the brochure condition. With respect to test utilization, only 2 of the 29 (7%) app condition participants who ordered a test used it, versus 2 of 9 (22.2%) in the brochure condition, for ~3% test usage rate in each condition (2/70 = 2.9%, vs. 2/68 = 2.9%; app vs. brochure). The number of test users (n = 4 total) was too small for a meaningful statistical test.

Regarding participant characteristics, app users were more likely to order a test if they were White (RR = 3.766, *p* < 0.001) and were non‐users of e‐cigarettes (RR = 4.523, *p* < 0.001). The proportion of participants ordering tests was similar among students who lived on and off‐campus (27 vs. 30%, respectively). Table 1 shows the incidence rate of participants ordering a test by condition and participants' attributes. Among females, the likelihood of ordering a test in the app condition was appropriately twice that of the brochure condition (RR = 6.84, *p* < 0.001).

**TABLE 1:** Incidence of test ordering between radon app and brochure conditions by participant characteristics

	Ordered Test		Z	*p*	RR	95% CI LL	95% CI UL	Interaction *p*
	*n*	%						
Radon app	29	41.43	3.716	<0.001	3.129	1.604	6.103	
Brochure	9	13.23^b^						
Female								0.016
Radon app	21	47.73	4.251	<0.001	6.841	2.200	21.270	
Brochure	3	6.98						
Male								
Radon app	8	32	0.542	0.294	1.280	0.521	3.143	
Brochure	6	25						
White								0.463
Radon app	23	40.35	3.607	<0.001	3.766	1.660	8.545	
Brochure	6	10.71						
Other								
Radon app	6	50	1.116	0.132	1.833	0.599	5.611	
Brochure	3	27.27						
< $100 k								0.733
Radon app	13	44.83	2.714	0.003	3.885	1.245	12.127	
Brochure	3	11.54						
≥$100 k								
Radon app	8	33.33	1.757	0.039	3.167	0.760	13.204	
Brochure	2	10.53						
On‐campus living								0.420
Radon app	14	50	3.162	<0.001	2.561	1.035	6.337	
Brochure	4	12.5						
Off‐campus living								
Radon app	15	36.59	2.200	0.014	4.000	1.488	10.751	
Brochure	5	14.29						
Use tobacco^a^								0.971
Radon app	3	42.86						
Brochure	0	0						
No tobacco								
Radon app	26	41.94	3.493	<0.001	2.982	1.522	5.844	
Brochure	9	14.06						
Use E‐cigarettes								0.122
Radon app	8	47.06	0.559	0.288	1.294	0.511	3.281	
Brochure	4	36.36						
No e‐cigarettes								
Radon app	21	40.38	3.820	<0.001	4.523	1.840	11.117	
Brochure	5	8.93						

^a^ Too few participants in groups to make valid comparisons.

^b^ Although 10 out of 68 brochure recipients ordered radon tests, one did not report sex and thus was not useable in inferential analyses.

## 4 DISCUSSION

We compared the radon app versus printed brochures on radon knowledge, attitudes, efficacy perceptions, and radon testing. Increases in radon knowledge and self‐efficacy were significantly greater among app users. Radon app participants requested tests at three time the rate of brochure participants (41 vs. 13%). These findings are consistent with findings demonstrating the superiority of apps vs. traditional media in subjects as diverse as coronary heart disease and antibiotic use.^15^, ^16^

The radon app was markedly more effective than brochures in stimulating test requests, that is, in promoting actual change in behavior, not merely knowledge. The percentage of brochure participants who ordered tests (13%) was similar to that observed in a clinical trial in Minnesota that utilized brochures (14%).^3^ Females in the app condition were significantly more likely than males to order tests. This is consistent with several studies showing that the use of health‐related apps is more common among females.^4^, ^17^ Living on or off‐campus did not materially influence test requests (27 vs. 30% requesting tests, respectively). Test utilization rates were low regardless of experimental condition. This may relate to the fact that the majority of students lived on campus and may have believed that radon was not a threat in university housing.

Our study has several limitations. Firstly, approximately 23% of participants were lost to follow‐up. Our losses to follow‐up are consistent with average attrition rates (18%) in trials of health behavior change.^18^ Loss was greater in the brochure condition (26.5% vs. 18.6%), which may reflect the greater interactivity of the app. Secondly, results from university students may not be generalizable to other populations. We considered North Dakotan undergraduates to be an appropriate population in light of North Dakota's high radon levels and because the age of many undergraduates resembles that of first‐time home‐buyers, a target population for radon education efforts.^9^, ^19^ The fact that most participants lived in university housing also is a limitation. Lastly, the app installation was completed in a guided setting, which is unlike settings in which individuals typically download apps. However, there are settings where guided installation could be employed, for example, Tobacco Quitlines. Quitline clients may be particularly motivated to test their homes given that radon potentiates the effect of smoking on lung cancer 10‐fold.^20^ We are currently testing the app among adult parents of young children, another population that is especially sensitive to radon health messages.^21^

In summary, the radon app markedly outperformed printed brochures in increasing radon knowledge and requests for tests among university students. Future development of the app should focus on additional, adult populations and on methods to increase test utilization.

## AUTHOR CONTRIBUTIONS

Conception: Soojung Kim and Gary G. Schwartz, Intervention: Soojung Kim and Tiffany Chiu, Writing: Soojung Kim, Marilyn G. Klug, and Gary G. Schwartz, Statistical analysis: Marilyn G. Klug, Editing and review: Soojung Kim, Tiffany Chiu, Marilyn G. Klug, David Schmitz, and Gary G. Schwartz.

## FUNDING INFORMATION

Research reported in this publication was supported by the National Institute of General Medical Sciences of the NIH Under Award Number U54GM128729 and by a pilot grant from that award to SK.

## CONFLICT OF INTEREST

None of the authors have any conflict of interest with regard to this research.

## PATIENT CONSENT

All participants completed and signed a standard consent form via computer. This project was approved by the IRB of the University of North Dakota School of Medicine & Health Sciences which considered it “expedited”, IRB # IRB0003245.

The trial was registered at **ClinicalTrials.gov, with ID#: NCT04980521**.

## Data Availability

Data generated from this study are not publicly available but would be made available upon reasonable request to the senior author.
